# Graphene quantum dots as cysteine protease nanocarriers against stored grain insect pests

**DOI:** 10.1038/s41598-020-60432-5

**Published:** 2020-02-26

**Authors:** Muazzama Batool, Dilshad Hussain, Ahmed Akrem, Muhammad Najam-ul-Haq, Shafqat Saeed, Syed Muhammad Zaka, Muhammad Shoib Nawaz, Friedrich Buck, Qamar Saeed

**Affiliations:** 1 Department of Entomology, Faculty of Agricultural Sciences & Technology, Bahauddin Zakariya University, 60800 Multan, Pakistan; 2 Department of Biochemistry, Institute of Chemical Sciences, Bahauddin Zakariya University, 60800 Multan, Pakistan; 3 Botany Division, Institute of Pure & Applied Biology, Bahauddin Zakariya University, 60800 Multan, Pakistan; 4 Department of Entomology, Muhammad Nawaz Sharif University of Agriculture, 60000 Multan, Pakistan; 5 Soil and Environmental Biotechnology Division, National Institute of Biotechnology and Genetic Engineering, 44000 Faisalabad, Pakistan; 6 Institute of Clinical Chemistry, University Medical Centre Hamburg-Eppendorf, Hamburg, Martinistr, 52, 20246 Germany

**Keywords:** Proteases, Plant sciences

## Abstract

Storing grains remain vulnerable to insect pest attack. The present study developed a biopesticide using biomolecules and their encapsulation in nanoparticles. A 25 kDa cysteine protease extracted from seeds of *Albizia procera* (*Ap*CP) was encapsulated in graphene quantum dots (GQDs). The insecticidal activity of *Ap*CP, with or without GQDs, against two stored grain insect pests, *Tribolium castaneum* (Herbst) and *Rhyzopertha dominica* (Fabricius) was explored. Insects were exposed to three concentrations 7.0, 3.5 and 1.7 mg of *Ap*CP per a gram of wheat flour and grains. The insecticidal activity of *Ap*CP encapsulated with GQDs was improved compared to that of *Ap*CP without GQDs for both insect pests. The number of eggs and larvae of *T. castaneum* was reduced by 49% and 86%, respectively. Larval mortality was increased to 72%, and adult eclosion of *T. castaneum* was reduced by 98% at a 7.0 mg/g concentration of *Ap*CP with GQDs compared to that of *Ap*CP without GQDs. Exposure to 7.0 mg/g *Ap*CP with GQDs, the number of *R. dominica* eggs and larvae was reduced by 72% and 92% respectively, larval mortality was increased by 90%, and eclosion was reduced by 97%. The extraction, purification, characterization, quantification and encapsulation of *Ap*CP with GQDs were also studied. Cysteine protease nanocarriers have the potential to control stored grain insect pests.

## Introduction

According to an estimate, 25% of food worldwide is damaged as post-harvest losses, out of which 20% of the damage is caused by insect pests through discoloration, change in flavour, weight loss, fungal infestation, reduced nutritional value, and poor germination^1^. The moisture level in the reserve rises due to higher infestation, and grains become heated, causing hot spots. Insects move from hot spots to cooler areas for egg laying, and fungus develops in the grain stock, which causes a decrease in grain quality^2,3^.

Food can be secured by using crop protection chemicals and pesticides^4^. These chemicals minimize production losses caused by insect pests, weeds and microbial diseases^5^. Pesticides are a low-cost, fast and efficient source of pest control^6^. However, they are toxic and pollute the environment. Drawbacks also include human intoxication^7^ and chronic diseases such as cancer, asthma, diabetes, leukaemia, endocrine disorders, and Parkinson’s disease^8–10^. Synthetic pesticides can be substituted by botanical pesticides for which research is in progress^11^. Biopesticides are biodegradable, safe for non-target organisms and high-yield and they may replace conventional pesticides^12^. Biomolecules such as plant proteins have the insecticidal activity and can be used to develop biopesticides. They are encapsulated in nanocarriers to maximize the molecular absorption to target sites^13–15^. Cysteine proteases are proteolytic enzymes involved in chitin degradation of the exoskeleton and peritrophic matrix in the midgut in insects (such as in the cowpea weevil), resulting in mortality^16^. Catalytic residues of proteolytic activity are Cys-25, His-159, and Asn-175^17^.

Nanotechnology has redefined medicine, engineering, agriculture, the food industry, cosmetics and other fields of science^18–21^. Nanomaterial synthesis has given rise to quantum dots, which are tiny particles of a few nanometres in size. They are central in nanotechnology and are also called artificial atoms^22^. Quantum dots are photostable, optically sensitive and can be easily traced. Different sizes of quantum dots are responsive to different frequencies of light^23^. In agriculture, quantum dots are being applied as nanobiosensors in the detection of plant diseases, quality control of food, and monitoring of food additives, mycotoxins, microbial infestation, allergens, pesticide residues and precision farming^23–26^. Graphene-based quantum dots assume novel chemical/physical properties. GQDs (graphene quantum dots) also show low cytotoxicity, excellent solubility, stable photoluminescence, and better surface grafting and pose no environmental hazards as do toxic metal-based quantum dots, instead exhibiting eco-friendly behaviour^27,28^. Silica- and metal-based nanoparticles are also used to repel and control insect pests^29^. In the present study, *Albizia procera* cysteine protease (*Ap*CP) was encapsulated in graphene quantum dots (GQDs) to increase its insecticidal activity and adsorption to the target site. The formulation was tested against two stored grain insect pests. *Ap*Cp encapsulation with GQDs provides biopesticides with directed delivery and environmental safety.

## Results

### *Ap*CP purification and identification

**Figure 1:**
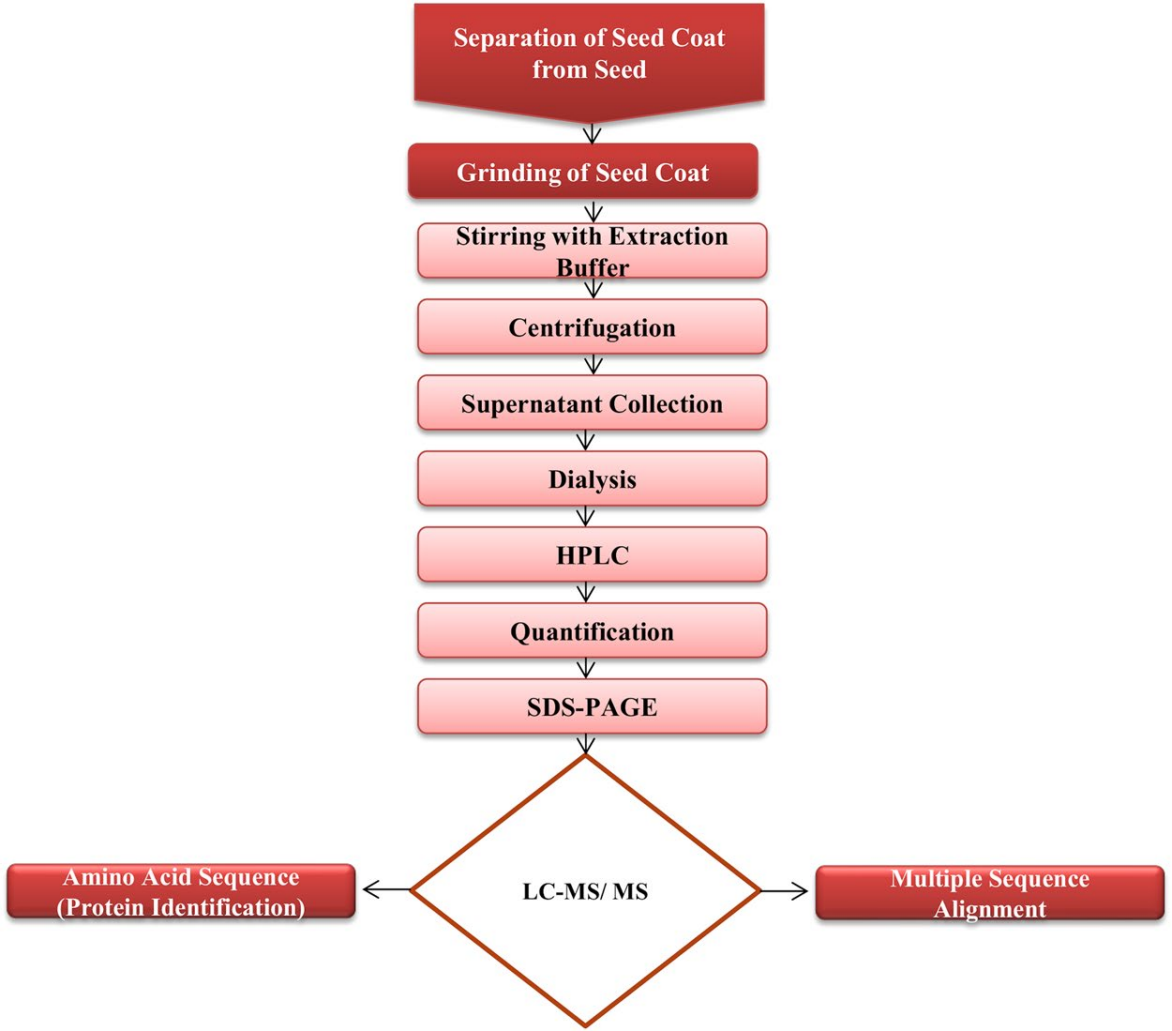
Schematic representation of *Ap*CP purification and identification.

The *A. procera* seed coat had a 25 kDa cysteine protease as a major protein constituent. The *Ap*CP sample was separated from the non-protein content by dialysis. SDS-PAGE analyses of reduced and non-reduced samples showed that *Ap*CP had no inter-chain disulfide linkages (Fig. S1). A high-intensity peak at a retention time of 13.9 minutes belonged to the cysteine protease extracted from *A. procera*. Few other peaks might correspond to the impurities in the sample; however, the chromatogram indicated that this protein was sufficiently pure and could be used against grain insects. The quantity of *Ap*CP was calculated as 4.7 mg/mL by a Nanodrop spectrophotometer. Figure 1 illustrates the steps for protein purification and identification.

**Figure 2:**
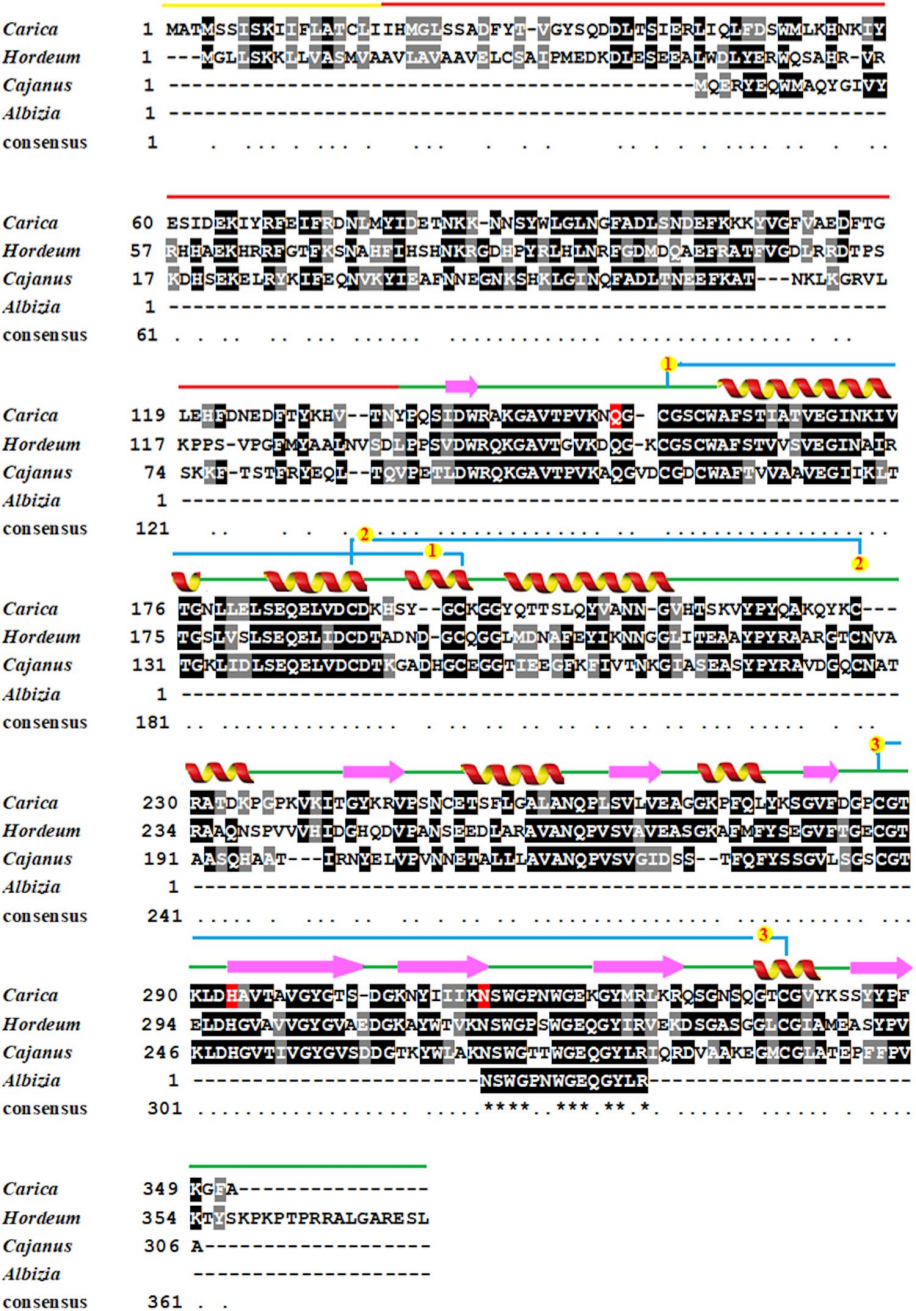
Multiple sequence alignment of the *Albizia procera* amino acid sequence with *Carica papaya, Hordeum vulgare* and *Cajanus cajan*. The signal peptide comprises eighteen amino acids marked by yellow bars, while the pro-peptide peptide ranges between 19 and 134 (red bars). Three intra-strand disulfide linkages are indicated by yellow circles and corresponding numbers. Catalytic residues (Gln, His and Asp) are conserved and indicated by a red background.

LC-MS/MS (Fig. S2) analysis produced an amino acid sequence (NSWGPNWGEQGYLR) that was subjected to BLAST in the online UniProtKB database. The sequence exhibited maximum sequence homology with vignain from *Cajanus cajan* cysteine protease (92.9%), followed by *Hordeum vulgare* EP-B2 (85.7%) and *Carica papaya* chymopapain (85.7%), as shown in Table S1 (supporting information). Multiple sequence alignments of *Ap*CP were prepared with closely related plant cysteine proteases (Fig. 2). Cysteine proteases (CPs) had a molecular mass range between 21–30 kDa. The best characterized family of cysteine proteases was that containing papain (PDB ID: 1YAL), possessing 8 alpha helices, 8 beta sheets and 17 loops in the molecular structure.

### Characterization of GQDs

**Figure 3:**
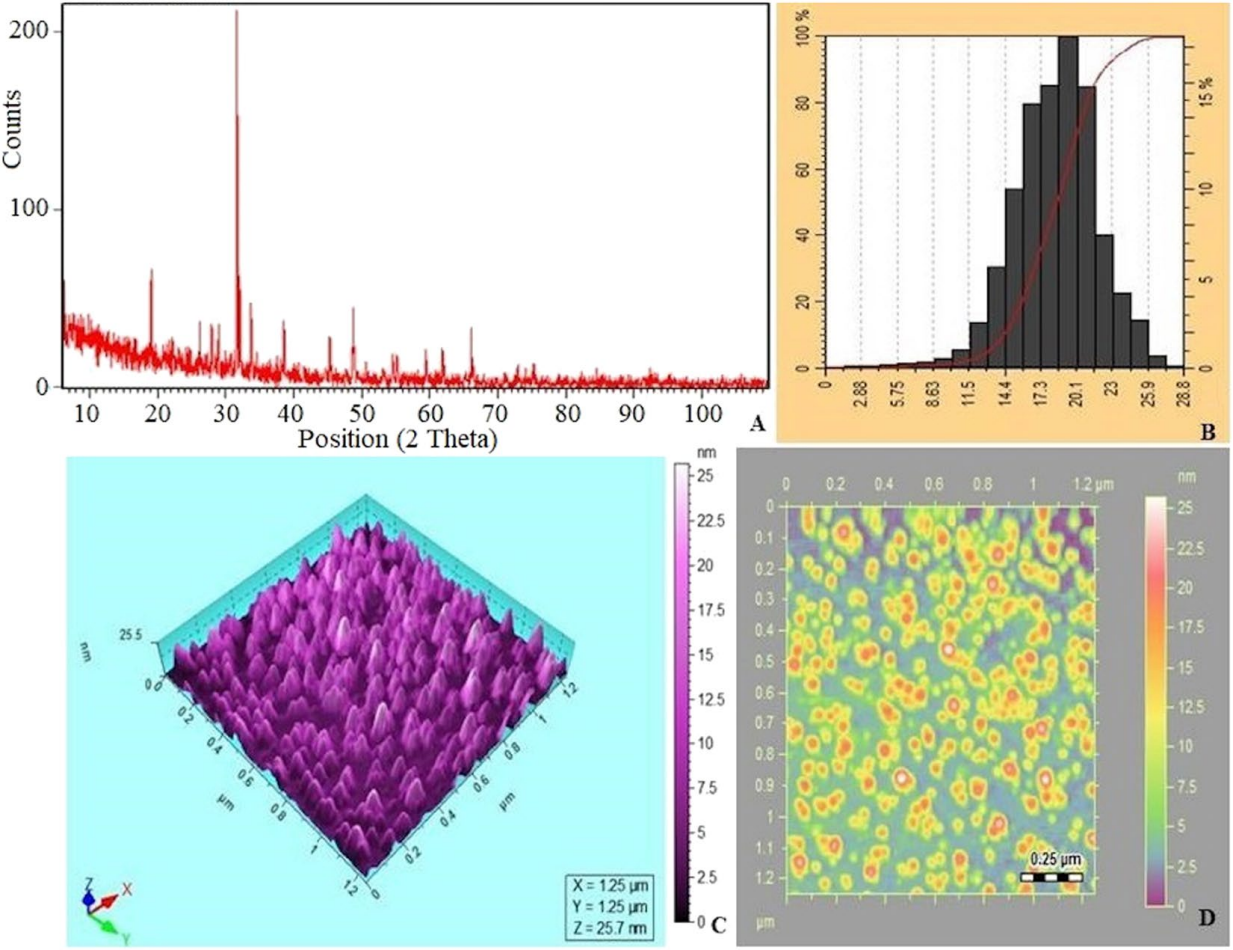
(**A**) XRD analysis of graphene quantum dots, (**B**) Particle size distribution profile showing ultra-small GQDs ranging from 13–23 nm with a maximum diameter up to 17 nm, (**C**) AFM image and (**D**) AFM heat map.

Attenuated total reflectance (ATR) characterization of graphene quantum dots indicated C=C skeletal vibrations of aromatic stretching, carboxyl groups and hydroxyl groups at 1684 and 3400 cm^−1^ (Fig. S3). There were absorption bands at 2961.37 cm^−1^ and 2889.70 cm^−1^ due to C-H stretching, and band at 1667.11 cm^−1^ corresponding to C = C aromatic stretching was observed. X-ray diffraction (XRD) analysis revealed the crystallinity and phase of the material. GQDs exhibited peaks at 19.11° and 32.09° for Graphene oxide and 28.51° for graphite. The peaks matched those listed in the database of JCPDS cards (1906–29). The particle size range was calculated as 17 nm from the X-ray diffraction results by the Scherrer equation (Fig. 3A). The particle size distribution was determined by atomic force microscopy (AFM) (Fig. 3B) and quantum dots exhibited particle size distribution ranging from 10 to 25 nm. The 3D view and heat map of atomic force microscopy indicate homogeneity of the particles and proper dispersion (Fig. 3C,D).

### Profiling for optimum activity conditions

*Ap*CP was extracted in phosphate buffer and distilled water to evaluate the best extraction and consequent maximum insecticidal activity. Extracts were tested against *T. castaneum* and *R. dominica*, and eggs, larvae, larval mortality and adult eclosion were evaluated. Insecticidal activities of *Ap*CP extracted by phosphate buffer and distilled water were not significant for *T. castaneum* eggs (F = 0.00, df = 2,6, P = 0.9967), larvae (F = 0.12, df = 2,6, P = 0.8902), larval mortality (F = 1.43, df = 2,6, P = 0.3116) and adults (F = 0.12, df = 2,6, P = 0.8859).

**Figure 4:**
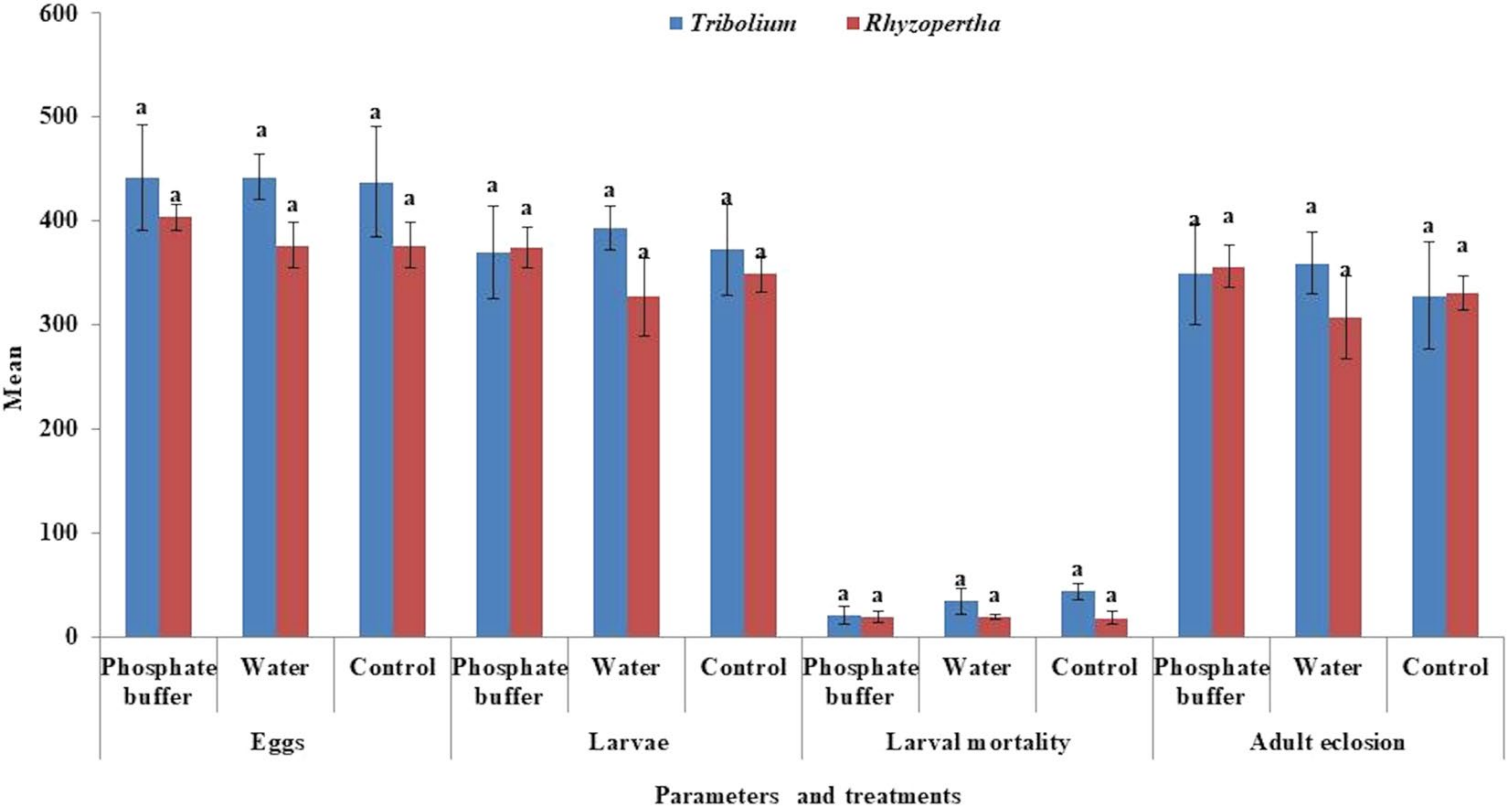
Optimization of *Ap*CP insecticidal activity against *T. castaneum* and *R. dominica* by feeding bioassays. Different letters above bars indicate significant differences.

The effects of *Ap*CP extracted by phosphate buffer and distilled water on *R. dominica* eggs (F = 0.67, df = 2,6, P = 0.5462), larvae (F = 0.83, df = 2,6, P = 0.4790), larval mortality (F = 0.02, df = 2,6, P = 0.9794) and adults (F = 0.78, df = 2,6, P = 0.5017) were also similar as control without *Ap*CP (Fig. 4). The *Ap*CP has no optimum activity to these two stored gran insect pests under phosphate buffer and water extraction conditions.

### Contact toxicity of *Ap*CP with and without GQDs

The optimized amount of GQDs for encapsulation of papain was 0.05 g when shaken at 30 ± 2 °C in solution (Fig. S4, Table S2). *Ap*CP encapsulated in GQDs under the same conditions was implemented for the contact and feeding bioassays. Residual toxicity data (Tables 1 and 2) of *Ap*CP (with and without GQDs) against *T. castaneum* after 24 hours of treatment showed that the LC_50_ value with encapsulation was 0.759 mg/L and it was 0.894 mg/L without encapsulation. After 48 hours, the LC_50_ values were 0.620 mg/L (with encapsulation) and 0.827 mg/L (without). Similar toxicity was observed after 72 hours of treatment, i.e., LC_50_ values of 0.577 mg/L and 0.634 mg/L with and without encapsulation, respectively. Moreover, GQDs encapsulation did not affect the LC_50_ of *Ap*CP to *T. castaneum* (Tables 1 and 2).

**Table 1:** LC_50_ of *Ap*CP without encapsulation of GQDs for *T. castaneum* and *R. dominica*.

Insect	Hours after treatment	LC_50_ (95% FL) (mg/L)	LC_90_ (95% FL) (mg/L)	Slope (±S.E)	X2*	df**	P
Tribolium	24	0.894 (0.738–1.084)	1.323 (1.092–1.602)	7.576	0.719	1	0.3964721
	48	0.827 (0.668–1.024)	1.321 (1.067–1.635)	6.376	0.631	1	0.4269887
	72	0.634 (0.385–0.742)	1.175 (0.846–1.632)	3.909	0.691	1	0.4058245
Rhyzopertha	24	—	—	—	—	—	—
	48	0.724 (0.585–0.897)	1.173 (0.947–1.454)	6.941	0.445	1	0.504719
	72	0.501 (0.367–1.384)	1.014 (0.743–1.384)	4.46	0.693	1	0.4051458

*Chi square, **degree of freedom.

**Table 2:** LC_50_ of *Ap*CP with encapsulation of GQDs for *T. castaneum* and *R. dominica*.

Insect	Hours after treatment	LC50 (95% FL) (mg/L)	LC90 (95% FL) (mg/L)	Slope (±S.E.)	X2*	df**	P
Tribolium	24	0.759 (0.559–1.032)	1.845 (1.359–2.507)	3.485	0.034	2	0.9831437
	48	0.620 (0.452–0.849)	1.565 (1.142–2.144)	3.363	0.035	2	0.9826522
	72	0.577 (0.430–0.774)	1.408 (1.049–1.889)	3.762	0.001	2	0.9995001
Rhyzopertha	24	0.771 (0.565–1.051)	1.913 (1.403–2.611)	3.418	0.029	2	0.9856046
	48	0.643 (0.481–0.862)	1.508 (1.126–2.019)	3.759	0.070	2	0.9656054
	72	0.449 (0.338–0.597)	0.993 (0.748–1.319)	4.239	0.033	2	0.9836354

*Chi square, **degree of freedom.

The contact toxicity data (Tables 1 and 2) of *Ap*CP against *R. dominica* after 24 hours of treatment showed that the LC_50_ was 0.771 mg/L with encapsulation, and there was no lethal effects on insects without encapsulation. After 48 hours, the LC_50_ values were 0.643 mg/L (with encapsulation) and 0.724 mg/L (without). Similar toxicity was observed after 72 hours of treatment, i.e., LC_50_ values of 0.449 mg/L and 0.501 mg/L with and without encapsulation, respectively. The toxicities after 24, 48 and 72 hrs were similar (95% FL overlapped with each other). Encapsulation of GQDs was responsible for a slight change in LC_50_, but the results remained not significant.

### Feeding toxicity of *Ap*CP with and without GQDs

#### *T. castaneum*

**Figure 5:**
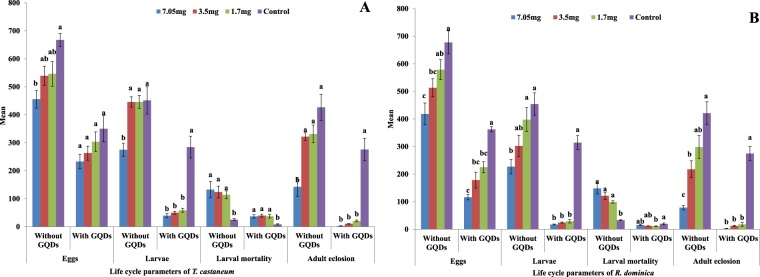
Response of *T. castaneum* (**A**) and *R. dominica* (**B**) to cysteine protease with and without GQDs. Different letters above bars indicate significant differences.

The primary and secondary stored grain insect pest *T. castaneum* was subjected to *Ap*CP alone or encapsulated in GQDs. The number of eggs of *T. castaneum* at 7.0, 3.5 and 1.7 mg/g *Ap*CP, with and without GQD encapsulation, was concentration dependent, i.e., the highest concentration exhibited the lowest number of eggs. *Ap*CP encapsulated in GQDs resulted in a statistically lower number of eggs (232.8 ± 25.6, F = 6.59, df = 3,16, P = 0.0041), in contrast to that of *Ap*CP treatments without GQDs (455.8 ± 31.5) at maximum concentration. Moreover, the control had 667.6 ± 23.1 eggs. The *Ap*Cp insecticidal efficacy increased by 49%, 51% and 45.4% for the three tested concentrations with encapsulation compared to that of *Ap*CP without encapsulation (Fig. 5A).

Larvae of *T. castaneum* at three concentrations of *Ap*CP with GQDs were observed to be statistically fewer in number (39.4 ± 7.4) at high concentrations of *Ap*CP (F = 33.6, df = 3,16, P < 0.0001), whereas the highest number of larvae was found in the control (284.4 ± 39). *Ap*CP treatments without GQD encapsulation induced a significant decrease in larvae with increasing concentration (F = 7.77, df = 3,16, P = 0.0020). However, in treatments without encapsulation treatments, larvae were high in number (275.2 ± 23.4) compared to those in encapsulated treatments at the same concentrations. The difference in insecticidal efficacy (in terms of larvae count) between encapsulated and non-encapsulated *Ap*CP at the three tested concentrations was 85.7%, 80% and 76.8%, respectively (Table 3).

**Table 3:** Percent increase in insecticidal efficiency of *Ap*CP after encapsulation with GQDs.

Conc (mg/g)	*T. castaneum*				*R. dominica*			
	Eggs	larvae	larval mortality	adult eclosion	Eggs	larvae	larval mortality	adult eclosion
**7**	49% ± 4.4	85.7% ± 1.9	72.8% ± 6.3	98.2% ± 0.9	72.2% ± 3.4	92.4% ± 0.7	89.8% ± 1.9	97.3% ± 1.2
**3.5**	51% ± 3.6	80% ± 1.2	68% ± 7.5	96.7% ± 0.2	65.3% ± 5.4	92% ± 1.0	90% ± 1.6	94.4% ± 1.0
**1.7**	45.4% ± 8.9	76.8% ± 2.2	67% ± 5.6	93.7% ± 1.3	61.2% ± 5.2	92% ± 1.0	88.8% ± 1.3	94% ± 1.5

Statistically higher larval mortality was recorded in treatments of *Ap*CP with GQDs compared to treatments of *Ap*CP without GQDs at all concentrations. All the tested concentrations tended to show significant larval mortality (F = 7.88, df = 3,16, P = 0.0019). Larval mortality (when comparing the encapsulated and non-encapsulated *Ap*CP) increased by 72.8%, 68% and 67% at the three concentrations, respectively (Table 3).

The statistically lowest population build-up of adult eclosion was recorded in treatments with *Ap*CP encapsulated with GQDs. The control treatment showed the statistically highest adult eclosion (275 ± 40.3) compared to that of the *Ap*CP treatments (F = 42.8, df = 3,16, P < 0.0001). The adult eclosion of the insects decreased by 98.2%, 96.7% and 93.7% for the three concentrations of *Ap*CP with GQD encapsulation compared to that for the three concentrations of *Ap*CP alone (Table 3).

#### *R. dominica*

When *R. dominica* was fed on a diet admixed with *Ap*CP with GQD encapsulation at three concentrations (7.0, 3.5 and 1.7 mg/g) or fed a control diet without *Ap*CP, the results of eggs number were statistically significant (F = 31.7, df = 3,16, P < 0.0001). The number of eggs of *R. dominica* in the highest concentration and in the control was 116.4 ± 9.6 and 362.5 ± 9.2, respectively. The feeding toxicity of the same three concentrations of *Ap*CP without encapsulation against *R. dominica* also revealed significant differences (F = 8.38, df = 3,16, P < 0.0001) from those of treatment with encapsulated *Ap*CP. At the highest concentration, the lowest number of eggs were recorded (418.2 ± 37.9), while 513 ± 32.9 eggs were found at the 3.5 mg/g concentration. The highest number of eggs were documented at the lowest concentration (578 ± 36.1), which was similar to that of the control (677.6 ± 41.6). Insects laid 72.2%, 65.3% and 61.2% fewer eggs at the three concentrations in decreasing order when exposed to *Ap*CP encapsulated with GQDs (Table 3).

*R. dominica* larvae number was significantly different between the *Ap*CP encapsulated in GQDs at the three concentrations and the control (F = 121, df = 3,16, P < 0.0001). Larvae were minimal at the highest concentration (17.4 ± 1.7), and the maximum was observed in the control (314.2 ± 25.6). Treatment with 7.0 mg/mL *Ap*CP without encapsulation resulted in 227 ± 26.3 larvae, and 454 ± 40.5 larvae were observed in the control. The insecticidal efficacy of *Ap*CP after GQD encapsulation was improved by 92.4%, 92% and 92% at the three tested concentrations in order of decreasing concentration (Table 3).

Statistically higher larval mortality was recorded for the encapsulated 7.0 mg/g *Ap*CP treatment in GQDs (148.4 ± 20.4), and low mortality (33 ± 1.8) was observed in the control (F = 16.3, df = 3,16, P < 0.0001). Larval mortality increased by 89.8%, 90% and 88.8% at the three concentrations of *Ap*CP encapsulated with GQDs compared to that for *Ap*CP without GQDs (Table 3).

Adult eclosion of the insects in the treatment with three tested concentrations of *Ap*CP in GQDs was recorded as 2.2 ± 0.9, 12.4 ± 2.1 and 17.8 ± 7.1. Treatments with *Ap*CP in GQDs led to significant differences in the maximum adult eclosion (274.6 ± 25.6) (F = 114, df = 3,16, P < 0.0001). Statistically higher adult eclosion of *R. dominica* was recorded in *Ap*CP without GQD encapsulation treatments (F = 18.5, df = 3,16, P < 0.0001). Adults were 78.6 ± 8.2 in number, in contrast to 2.2 ± 0.9 (in encapsulated treatments) at 7.0 mg/g. *Ap*CP encapsulated with GQDs tended to show 97.3%, 94.4% and 94% less adult eclosion at the three tested concentrations in decreasing order (Fig. 5B).

### Feeding toxicity of GQDs

**Figure 6:**
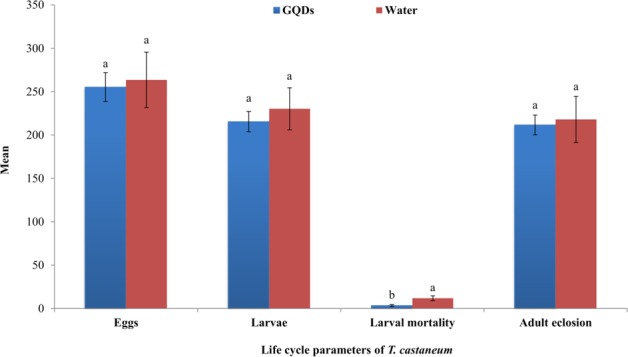
Feeding toxicity assay of GQDs to *T. castaneum* showing inert behaviour of GQDs. Different letters above bars indicate significant differences.

Wheat flour was admixed with GQDs and fed to *T. castaneum* to evaluate their effects on the life cycle. GQDs were inert regarding the life of red flour beetles, as explained by the results in Fig. 6. The results for the GQDs treatment and the control were similar for eggs (F = 0.05, df = 1,8, P = 0.8218), larvae (F = 0.30, df = 1,8, P = 0.5968) and eclosion (F = 0.05, df = 1,8, P = 0.8304). Water-treated units had higher mortality than GQD-treated units (F = 7.93, df = 1,8, P = 0.0226).

### Feeding toxicity of papain

**Figure 7:**
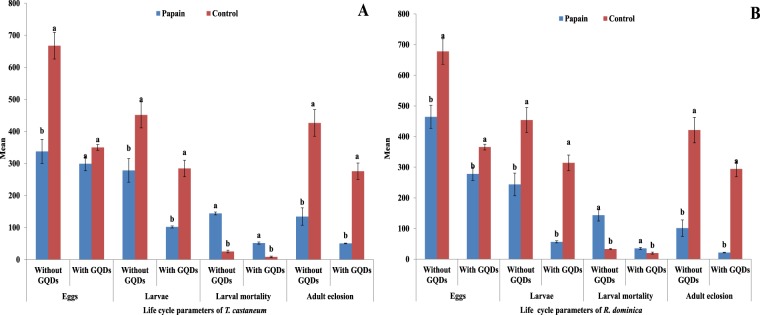
Response of *T. castaneum* (**A**) and *R. dominica* (**B**) to Papain with and without GQDs. Different letters above bars indicate significant differences.

Papain encapsulation with and without GQDs against *T. castaneum* exhibited statistically significant results in terms of eggs, larvae, larval mortality and adult eclosion. The insecticidal efficiency of papain after encapsulation with GQDs improved by 11.4% in terms of eggs, 63.3% in terms of larvae, 64.4% in terms of larval mortality and 37.6% in terms of adult eclosion compared to without GQDs (Fig. 7A).

The encapsulation of papain in GQDs as a treatment against *R. dominica* resulted in statistically fewer eggs and larvae and less adult eclosion compared to those after treatment without GQD encapsulation and those in the control. The insecticidal activity of papain increased by 40.1% in terms of eggs, 76.9% in terms of larvae, 75.6% in terms of larval mortality and 78.7% in terms of eclosion after encapsulation with GQDs (Fig. 7B).

## Discussion

The present study reported the insecticidal activity of cysteine protease derived from *A. procera* seed coat. The seed coat is a source of defensive chemicals for plants and serves as a physical barrier to intruders^30,31^. Soybean seed coat has been shown to be protective against the penetration of *Callosobruchus maculatus* into the seed^32^. *C. maculatus* larvae were tested against seed coat proteins from *A. lebbeck* by mixing these proteins with feed, similar to this study in that it also involves proteins mixed into feed. The papain-like cysteine protease was efficient against female oviposition and larval survival at 1%, 2% and 3% concentrations^16^, which were higher than the *Ap*CP concentrations of 7.0, 3.5 and 1.7 mg of *Ap*CP per a gram of wheat flour. This study also confirmed the effect of *Ap*Cp on oviposition (eggs) larval count (larvae) and larval mortality of treated insects.

**Figure 8:**
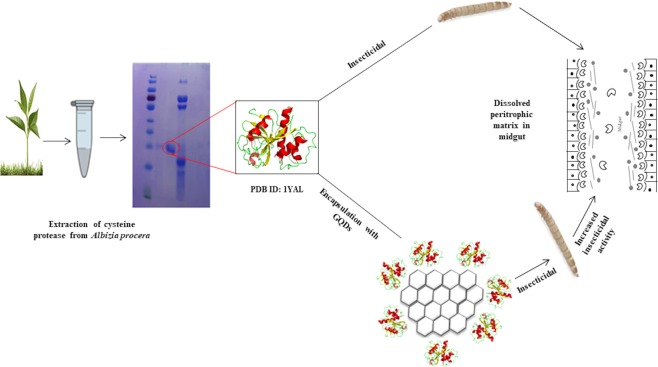
Graphical representation of cysteine protease encapsulation on GQDs for wheat grain insects.

The 25 kDa *Ap*CP is promising against *T. castaneum* and *R. dominica*, which are the key pests of warehouse commodities worldwide. The insecticidal activity was tested for the life cycle span of insect pests. Protein-treated insect pests showed a reduction in life cycle parameters including eggs, larvae and ecloded adults. The control experiments showed maximum adult eclosion compared to protein-treated units. Graphene was chosen as a carrier for *Ap*CP molecules to target the basement membrane, chitin, of the exoskeleton and peritrophic membrane of the insect mid gut. Disruption of the peritrophic matrix may affect larval development and other processes such as digestion, digestive enzyme recycling and nutrient absorption^16,33–35^. Small-sized GQDs with encapsulated *Ap*CP can enter into extracellular spaces and/or spaces between the cell membrane, hydrolysing the proteinaceous content of chitin and disrupting normal biochemical processes, leading to insect death (Fig. 8).

Optical properties of GQDs are key for their practical use. With varying degrees of photoluminescence, excellent chemical stability, and low cytotoxicity, GQDs are perfect for use in biological applications such as bioimaging, drug delivery, biosensing, DNA cleavage and environmental monitoring^28^. Bioimaging is the most important application of GQDs currently being used in life sciences because GQDs show decent photoluminescence below pH 7 and have low toxicity to different cells in a broad concentration range^36^. The results ensured that GQDs have no toxicity to *T. castaneum*. GQD formulations with cysteine protease could be a promising pesticide/insecticide against stored product insect pests. *R. dominica* and *T. castaneum* had disrupted life cycles when subjected to *Ap*CP and papain. At the highest evaluated protein concentration, the population of *T. castaneum* was reduced in terms of larvae and ecloded adults when subjected to GQDs loaded with *Ap*CP compared to the population of *T. castaneum* treated with *Ap*CP without GQDs. The *R. dominica* larvae population was reduced to 17.4 when treated with *Ap*CP-loaded GQDs compared to the 227 larvae observed in the treatment of *Ap*CP without GQDs. At the highest evaluated protein concentration in both insects, the adult population of GQD-encapsulated *Ap*CP treatment decreased compared to the population treated by *Ap*CP without GQDs.

Cysteine proteases have a two-domain structure. The active site for substrate binding is present between the domains. The catalytic residues of cysteine protease are Cys-25, His-159 and Asn-175. These residues are evolutionarily preserved in cysteine proteases. Papain is mutated to have Gln-19 instead of Cys-25 as substrate binding amino acid (PDB ID 1YAL). The enzymatic activity of cysteine proteases is due to the catalytic dyad formed by cysteine and histidine residues, which exist as an ion pair (–S–…H+Im–) in the pH range of 3.5–8.0^37,38^. An intermediate, i.e., S-acyl-enzyme, is formed via nucleophilic attack of the thiolate group of the cysteine residue by the carbonyl of the hydrolysed peptide bond with the release of the C-terminal fragment of the cleaved product. In the next step, the water molecule reacts with the intermediate, and the N-terminal fragment is released, regenerating free papain to begin a new catalytic cycle^39^. The *Ap*CP amino acid sequence NSWGPNWGEQGYLR has maximum homology with *C. papaya*, *H. vulgare*, *and C. cajan*, which are well-studied cysteine proteases. Cysteine protease and its GQD formulation were also used for contact toxicity against the insect pests. The LC_50_ of proteins decreased after encapsulation of *Ap*CP with GQDs. The combination can thus be prescribed for surface treatment of storage structures to replace potentially hazardous insecticides such as organophosphates and carbamates. The results of parallel studies confirm that the *Ap*CP active site targets the peritrophic matrix of the insect mid gut, which is hydrolysed by cysteine protease. The formulation of *Ap*CP with GQDs would be a promising alternative to synthetic pesticides. Pesticides are widely applied during agricultural practices, which leads to human and environmental toxicity. Graphene quantum dots can be used to trace pesticide residues within the organisms, environment and food products. Graphene quantum dots could also be used for the formulation of environmentally safe pesticides and for targeted delivery of that formulation within the pest body. Commercial application of this research will help to mitigate the hazardous effect of synthetic pesticides on human health and the environment.

## Materials and Methods

### Insect culture

Insects *T. castaneum* and *R. dominica* were maintained at the Eco-Toxicology Laboratory in the Department of Entomology, Bahauddin Zakariya University, Multan, Pakistan (30°11′44 N; 71°28′31 E). Homogenous cultures of *R. dominica* and *T. castaneum* were maintained by rearing insects with wheat grains and flour, respectively, at 30 ± 5 °C and 60 ± 5% RH. Insects were separated by sex at the pupal stage by following the standard protocols^40,41^.

### Extraction of *Ap*CP

The seed coat of *A. procera* was separated, ground into a fine powder and suspended in Tris buffer (0.1 M, pH 7.0) with 0.15 M NaCl. The mixture was centrifuged at 5300 rpm for 15 min, and the supernatant was collected. Crude extract was dialysed (Spectra/Por 3, Catalogue No. 132724, MWCO 3 kDa) overnight with the same buffer to remove the salt from the extract. The extract was examined via SDS-PAGE by preparing one-dimensional 12% gels (E-VS10-SYS, omni PAGE Mini-System) to see the protein bands. Reduced *(β*-mercaptoethanol-treated) and non-reduced samples were heated at 95 °C for 5 min in a heating block to denature the samples. The gel was stained with Coomassie brilliant blue R-250 dye (CBBR-250) (Sigma Aldrich) to visualize the protein bands, and the molecular weights were determined by using Protein Ladder (Thermo Scientific™, Catalogue No. 26616).

### Purification and quantification of *Ap*CP

The protein sample was purified through acetone precipitation at a 1:5 (sample: acetone) ratio and centrifuged. The pellet was mixed in 1.0 M ammonium bicarbonate solution. Protein in the extract was determined by reverse phase HPLC using a Tecknochroma C18 column at a 60 min linear gradient of 0.1% TFA in water to 0.1% TFA in ACN at 0.1 mL/min. Protein was quantified by using a Nanodrop spectrophotometer.

### LC-MS/MS analysis

Protein bands stained with Coomassie dye were excised from the gel and reduced with dithiothreitol (0.005 M, 55 °C and 30 min). In-gel protein digestion was performed overnight with trypsin as per the described protocol^42^. Digested gel pieces were extracted with a 50% acetonitrile/5% formic acid solution and dried in a vacuum concentrator. LC-MS/MS measurements were made by loading samples on a nano-liquid chromatography (n-LC) system (Dionex UltiMate 3000) coupled via electrospray ionization (ESI) to an orbitrap mass spectrometer (Orbitrap Fusion, Germany). The protein solution was loaded on a trapping column (Acclaim PepMap C18; buffer A: 0.1% formic acid in H_2_O; buffer B: 0.1% formic acid in acetonitrile) with 2% buffer B, and peptides were eluted (200 mL/min). LC-MS/MS analysis was carried out in data-dependent acquisition (DDA) mode. The raw data were processed with Proteome Discoverer 2.0 (Thermo Scientific, Germany). For identification, MS/MS spectra were investigated with Sequest HT against *Arabidopsis* and the UniProtKB server, while identifications were manually validated.

### Contact toxicity bioassay

Petri dishes of 5 cm diameter were washed with distilled water and dried. Filter papers were cut into the same diameter. Three concentrations (7.0, 3.5 and 1.7 mg/mL) of *Ap*CP were made in Tris buffer solution and poured into beakers. Filter papers were immersed in the three tested concentrations for 30 sec with gentle agitation and then allowed to dry. Treated filter papers were set into petri dishes. Five pairs of newly born *T. castaneum* and *R. dominica* adults were placed in the petri dishes in five replicates. The mortalities were recorded after 6, 12, 24 and 72 hours of each concentration for both insects.

### Feeding toxicity bioassay

Three concentrations of *Ap*CP (7.0, 3.5 and 1.7 mg/mL) were prepared in Tris buffer. These concentrations were mixed with 150 g pre-sanitized wheat flour and grains for *T. castaneum* and *R. dominica*, respectively. The control was prepared with wheat flour and grains in Tris buffer. The treated flour, grains and control samples were spread thinly to dry in air. Wheat flour was ground into a fine powder. Treated flour and grains were divided into 5 replicates of 30 g in separate jars in each concentration of *Ap*CP. Each replicate/jar was supplied with 5 pairs of newly born *T. castaneum* and *R. dominica*. Treatment jars were placed at 30 ± 5 °C and 60 ± 5% RH. The adults of both insects were removed after one week. Treatment units were checked weekly to determine the changes in life cycle parameters of treated insects. Observations were recorded in terms of the number of eggs and larvae, larval mortality and adult eclosion of treated insects. *Ap*CP was extracted in 100 mL of pH 7 phosphate buffer and distilled water to check the maximum activity of protein. Feeding toxicity assays were conducted for the same life cycle parameters of both insects.

### GQD synthesis and insecticidal activity

Graphene quantum dots were prepared from graphite powder^43^ obtained by a refined Hummer’s method^44^. Two grams graphite powder was mixed with 120 mL sulfuric acid in an ice bath and stirred for 30 min. KMnO_4_ (11.6 g) was gradually mixed while heating at 30 °C for 2 hrs. Then, 80 mL deionized water was added, and the temperature was raised to 90 °C for 30 min. The temperature was lowered to 60 °C, and 160 mL deionized water was added. Next, 30% H_2_O_2_ was added until an orange-yellow solution was obtained. Then, 400 mL 5% HCl was mixed in, and the pH was maintained at 5 by washing through vacuum pump assembly. Blackish grey graphene oxide was obtained. One gram graphene oxide was agitated with 20 mL H_2_SO_4_ for 2 hrs in an ice bath. Afterwards, 10 mL 50% KMnO_4_ was added, the mixture was stirred for 2 hrs, and the temperature was raised to 50 °C for one hour. Then, 80 mL distilled water was added, and the temperature was increased to 90 °C for 30 min. After homogenization of KMnO_4_, 20 mL H_2_O_2_ was added to the ice bath, followed by distilled water until a yellow-brown solution appeared. The solution was ultrasonicated, and the pH was increased to 8 by adding NaOH. HCl was added to lower the pH to 4, giving a dark greenish-yellow mixture. Graphene quantum dots were characterized by Attenuated total reflectance (ATR), X-ray diffraction (XRD), Scherrer equation (www.intanano.com) and atomic force microscopy (AFM). Quantum dots were mixed with distilled water at concentrations of 0.5%, 0.25% and 0.12% and assessed for their contact and feeding toxicity to the stored grain insects using the methods discussed above.

### Encapsulation capacity of GQDs

Solutions of 100, 50, 40, 30, 20 and 10 ppm of commercial papaya latex cysteine protease (papain) (Sigma Aldrich, Catalogue No. P3375) were prepared from a 1000 ppm stock solution. A calibration curve of commercial papain was obtained using the standard solution. Solutions were mixed with quantum dots and shaken for 30 min, followed by filtration. Absorbance was recorded by using a UV-Visible spectrophotometer. Encapsulation was optimized by altering parameters such as the weight of GQDs, solution concentrations and temperature (below 50 °C). Different weights of GQDs (0.025, 0.1, 0.2 and 0.5 g) were used with a single concentration (100 ppm) at room temperature. Furthermore, a 100 ppm solution was mixed with 0.05 g GQDs at temperatures of 10, 20, 30, 40 and 50 °C. Similarly, 0.05 g GQDs were mixed with 10, 20, 30, 40 and 50 ppm papain solutions at room temperature. Encapsulation efficiency was calculated by the equation below:

$${\rm{Encapsulation}}\,{\rm{efficieny}}=\frac{{\rm{Initial}}\,{\rm{drug}}\,{\rm{conc}}.-{\rm{Conc}}.\,{\rm{of}}\,{\rm{free}}\,{\rm{drug}}\,{\rm{after}}\,{\rm{encapsulation}}}{{\rm{Initial}}\,{\rm{drug}}\,{\rm{conc}}.}\times 100$$

### Encapsulation of *Ap*CP and insecticidal assays

Encapsulation of *Ap*CP was performed with optimized conditions of loading capacity percentage, i.e., weight of GQDs, solution concentration and temperature. *Ap*CP encapsulated with GQDs was subjected to contact and feeding toxicity assays against two stored grain insect pests at the three above-mentioned concentrations by following the previously discussed methods. Papain is the most studied cysteine protease for its insecticidal activity and has a well-explained mode of action in the insect midgut of disrupting the peritrophic matrix. In this study, papain was also used (as a reference protein) to study its insecticidal effects with and without encapsulation of GQDs at a concentration of 3.5 mg/g. The hypothesis of increased insecticidal efficiency with the formulation of proteins with GQDs also proved to be successful for papain encapsulated in GQDs against tested stored grain insect pests.

### Data analysis

For the contact toxicity bioassay, insects were observed under 6, 12, 24, 48 and 72 hours for mortality and subjected to Probit^45^ analysis. For feeding toxicity assays, insects were observed weekly during their life cycle span to evaluate changes in the mean number of eggs and larvae, larval mortality and adult eclosion. Data were subjected to ANOVA, and homogenous groups were further subjected to Tukey’s HSD test through Statistix 8.1^46^.

## Supplementary information

Supporting information.
