# Supplementary material for: Graphene quantum dots as cysteine protease nanocarriers against stored grain insect pests
Source: Sci Rep. 2020 Feb 26;10:3444. doi: 10.1038/s41598-020-60432-5 (PMC7044290; doi:10.1038/s41598-020-60432-5)
Supplement: Supplementary file 1 — Supporting information. [file 41598_2020_60432_MOESM1_ESM.pdf]

Supplementary Information

For

Graphene quantum dots as cysteine protease nanocarriers against stored grain insect pests

Muazzama Batool[1], Dilshad Hussain[2,3], Ahmed Akrem[4], Muhammad Najam-ul-Haq[2], Shafqat Saeed[5], Syed Muhammad Zaka[1], Muhammad Shoib Nawaz[6], Friedrich Buck[7], Qamar Saeed[*1]

[*]Correspondence should be addressed to

Dr. Qamar Saeed

Department of Entomology, Faculty of Agricultural Sciences & Technology, Bahauddin Zakariya University, 60800 Multan, Pakistan.
Email: qamarsaeed@bzu.edu.pk

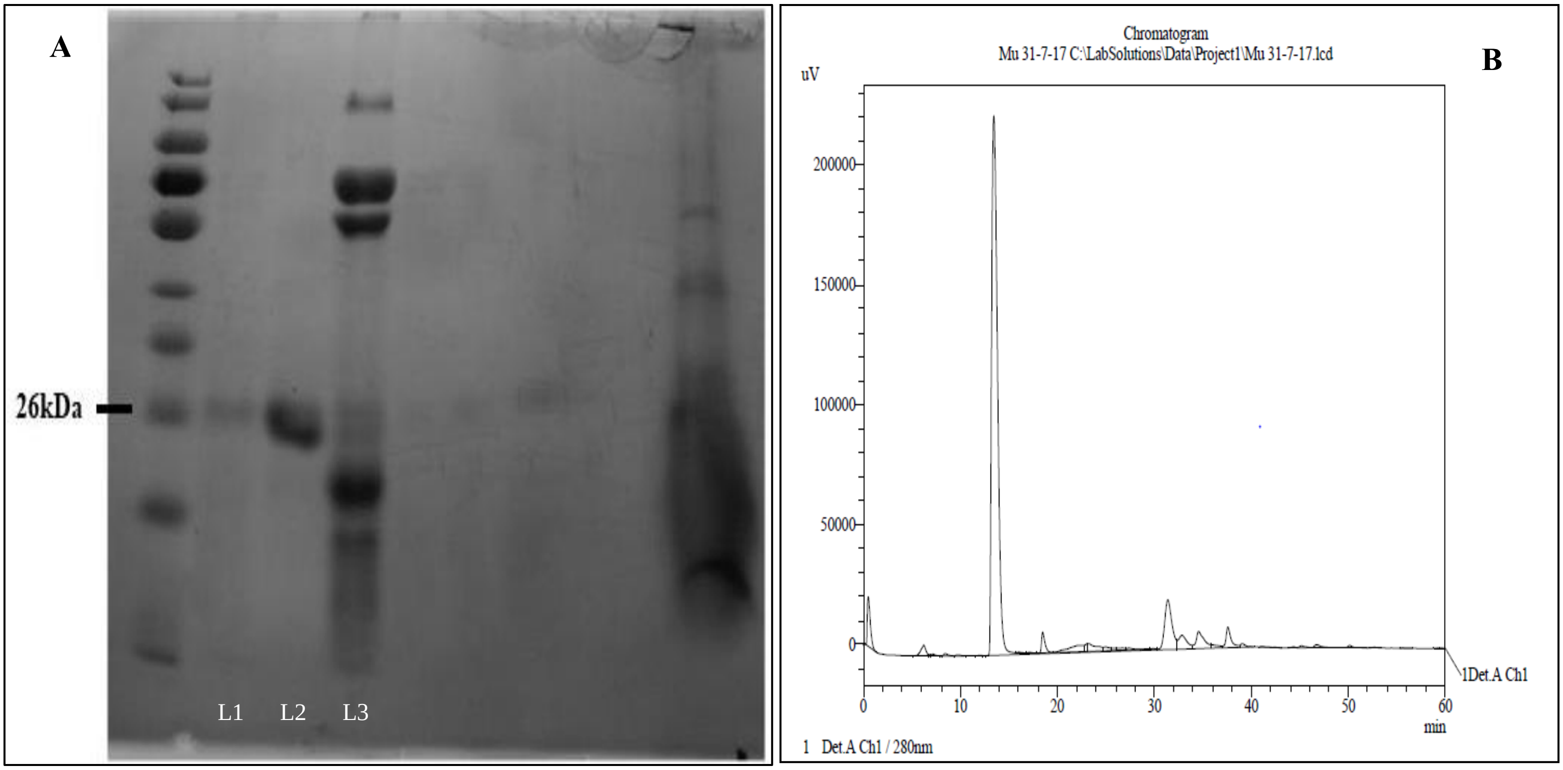

Figure S1: (A) Gel electrophoresis bands showing protein strands with 25kDa pure protein from seed coat (L1 and L2) and full seed proteins (L3) with protein weight marker of 26kDa (Thermo Scientific™, Catalogue No. 26616). Rest of the gel lanes are not related to this study. (B) HPLC chromatogram of cysteine protease from *Albizia procera.*

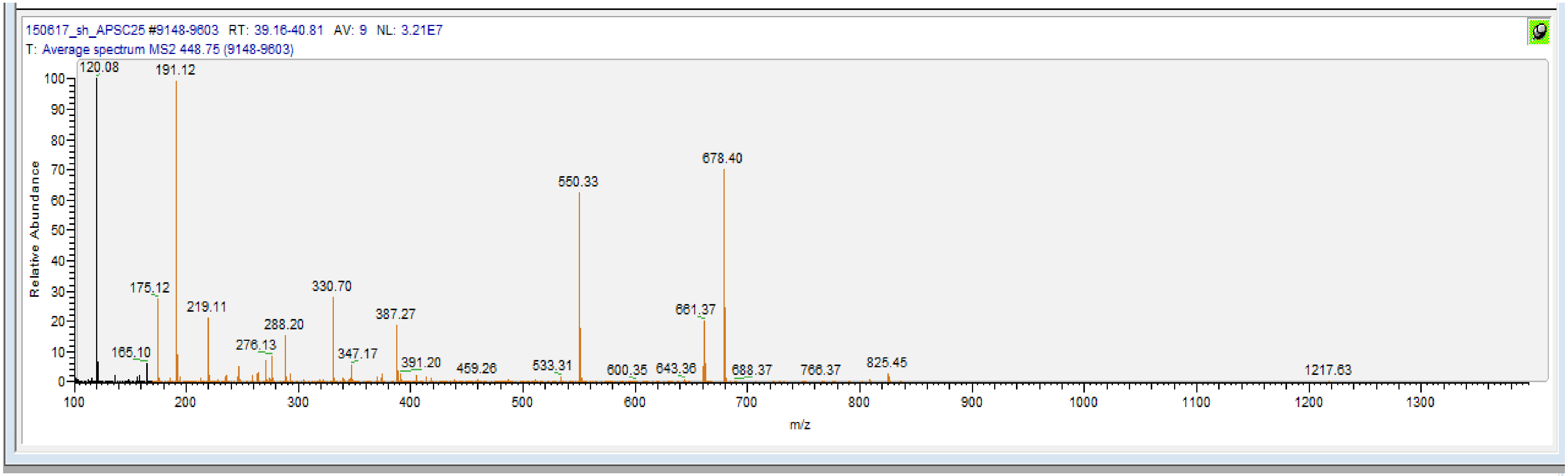
150617_sh_APSC25 #9148-9603 RT: 39.16-40.81 AV: 9 NL: 3.21E7
T: Average spectrum MS2 448.75 (9148-9603)
Relative Abundance
m/z
120.08
191.12
175.12
165.10
219.11
276.13
288.20
330.70
347.17
387.27
391.20
459.26
533.31
550.33
600.35
643.36
661.37
678.40
688.37
766.37
825.45
1217.63

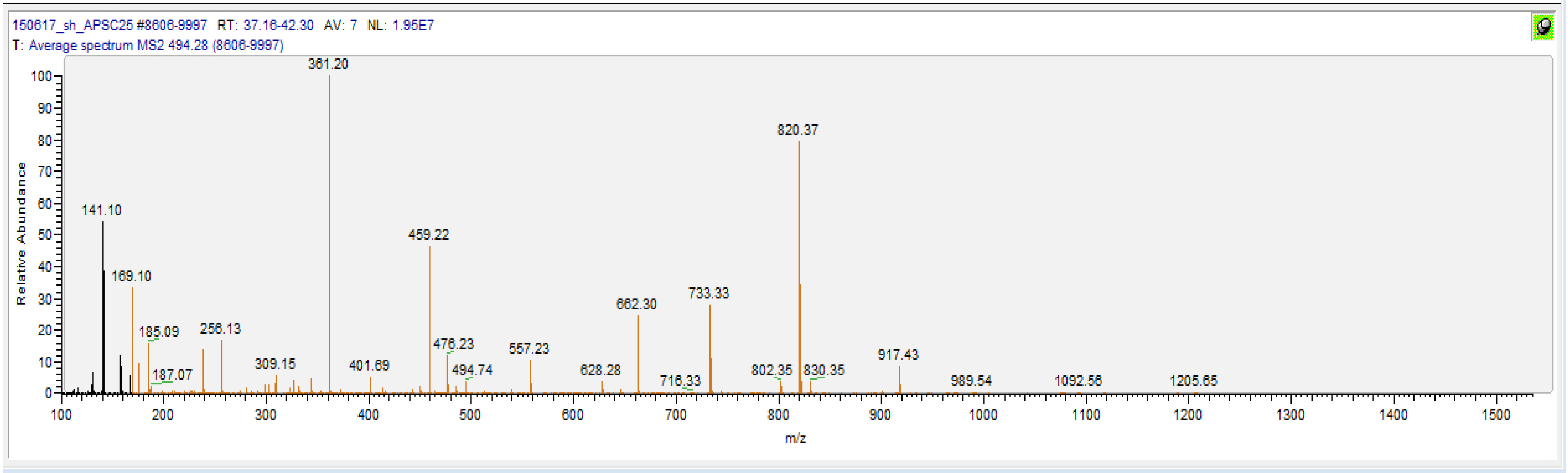
150617_sh_APSC25 #8606-9997 RT: 37.16-42.30 AV: 7 NL: 1.95E7
T: Average spectrum MS2 494.28 (8606-9997)
Relative Abundance
m/z
361.20
141.10
169.10
185.09
187.07
256.13
309.15
401.69
459.22
476.23
494.74
557.23
628.28
662.30
716.33
733.33
802.35
820.37
830.35
917.43
989.54
1092.56
1205.65

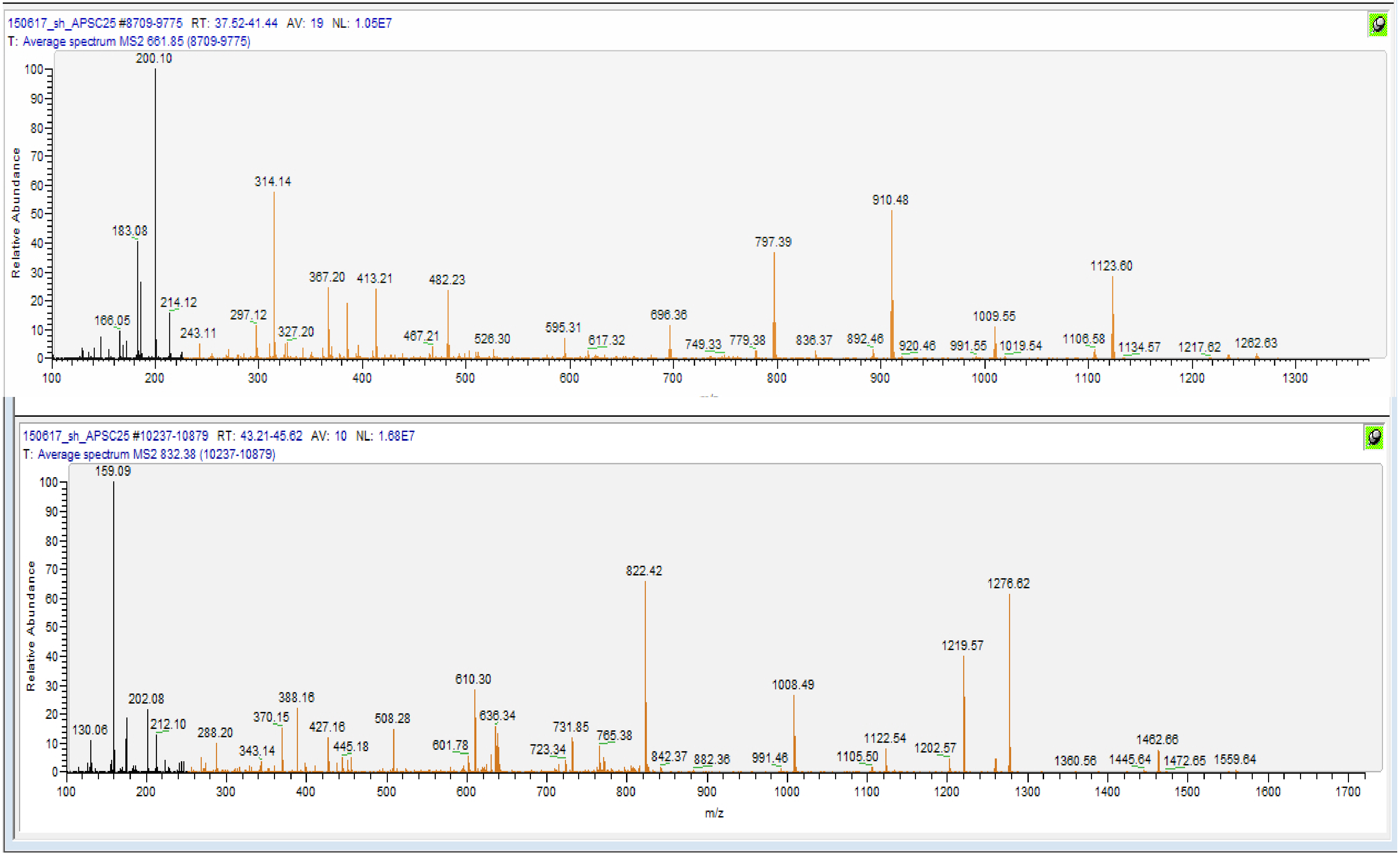

Figure S2: LC-MS/MS spectra of cysteine protease extracted from *Albizia procera* after gel purification and digestion, recorded at

different retention times.

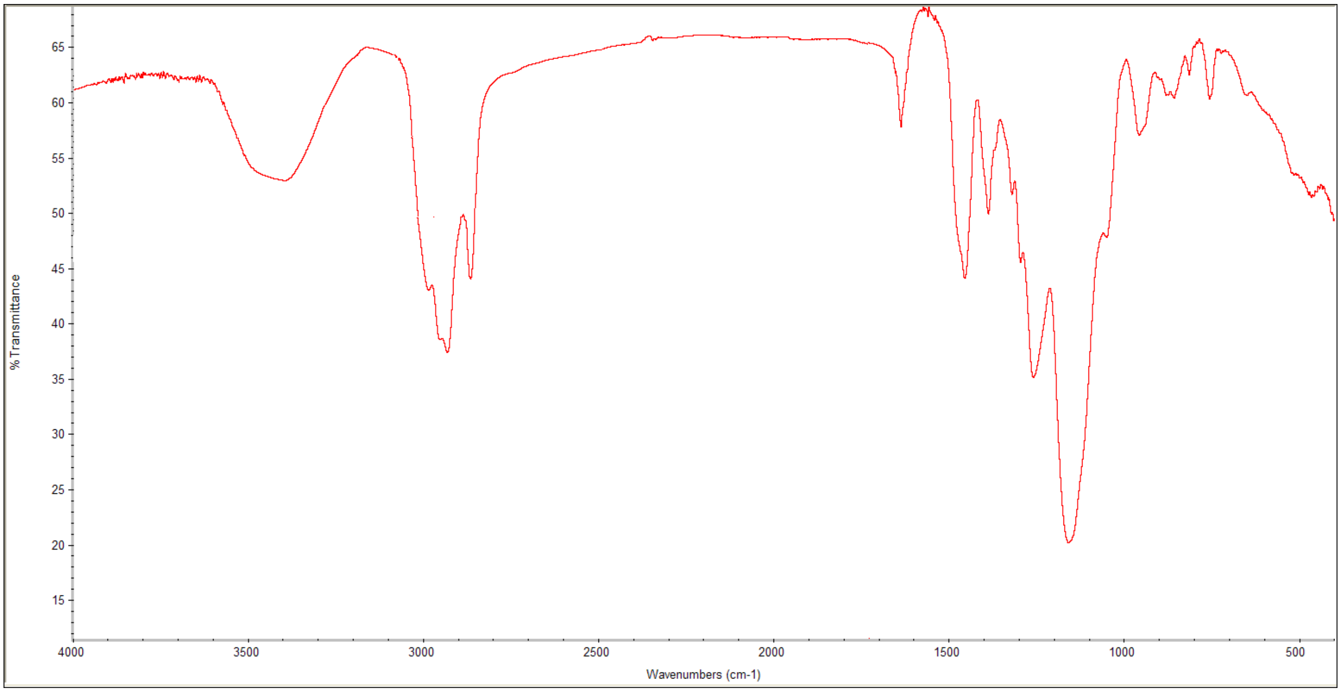

Figure S3: ATR spectrum of graphene quantum dots (GQDs)

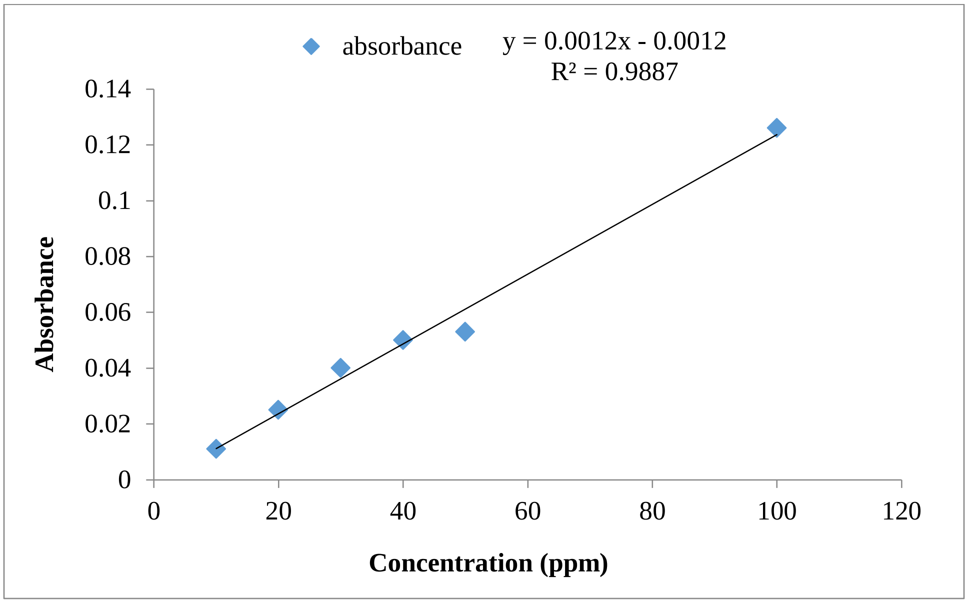

Figure S4: Calibration curve and optimization of papain loading to GQDs

Plants	Cysteine Protease	Amino Acid Sequence	Homology (%)	Reference (SwissProt)
Carica papaya	Chymopapain	NSWGPNWGE***K***GYMR	85.7	P14080
Hordeum vulgare	Cysteine Protinase EP-B2	NSWGP***S***WGEQGYIR	85.7	P25250
Cajanus cajan	Vignain	NSWG***TT***WGEQGYLR	92.9	A0A151TNH2
Albizia procera	Cysteine Protease	NSWGPNWGEQGYLR	100	This paper

Table S1: Amino acid sequence obtained from LC-MS/MS analysis and its homology comparison with other species.

Concentration (ppm)	Temperature ºC	Encapsulation effeciency (%)
100	10	65
100	20	62
100	30	68
100	40	68
100	50	65
**Concentration (ppm)**	**Weight of GQDs (g)**	**Encapsulation effeciency (%)**
100	0.025	45
100	0.05	70
100	0.1	65
100	0.2	62
**Concentration (ppm)**	**Weight of GQDs (g)**	**Encapsulation effeciency (%)**
10	0.05	10
20	0.05	25
30	0.05	40
40	0.05	62
50	0.05	60

Table S2: Encapsulation efficiency optimization at different parameters.
